# Protective Effects of Quinic Acid Against Disuse-Induced Skeletal Muscle Atrophy via Regulation of Inflammation and Oxidative Stress

**DOI:** 10.3390/foods14223833

**Published:** 2025-11-09

**Authors:** Mi-Bo Kim, Hyerin Lee, Junhui Kang, Bohkyung Kim, Jae-Kwan Hwang

**Affiliations:** 1 Department of Food Science and Nutrition, Pukyong National University, Busan 48513, Republic of Korea; mibokim1120@gmail.com; 2 Graduate Program in Bioindustrial Engineering, College of Life Science and Biotechnology, Yonsei University, Seoul 03722, Republic of Korea; hshs7915@naver.com (H.L.); junrim2577@naver.com (J.K.); 3 Department of Food Science and Nutrition, Pusan National University, Busan 46241, Republic of Korea; 4 BK21 FOUR Program, Precision Nutrition Program for Future Global Leaders, Pusan National University, Busan 46241, Republic of Korea

**Keywords:** quinic acid, disuse-induced muscle atrophy, L6 myotubes, inflammation, oxidative stress, protein turnover

## Abstract

Disuse-induced muscle atrophy (DMA), commonly resulting from immobilization, is driven by chronic inflammation and oxidative stress, which disrupts the balance between protein synthesis and degradation. Quinic acid (QA), a natural compound with known antioxidant and anti-inflammatory properties, was investigated for its potential to counteract muscle atrophy. Using a DMA-induced immobilization model in male C57BL/6N (8 weeks) mice, we found that oral QA administration significantly restored the weight and cross-sectional area of atrophic muscles and improved muscle function, as measured by grip strength and treadmill performance. QA also reduced the expression of pro-inflammatory cytokines (*Tnf*, *Il6*, and *Myostatin*) and E3 ubiquitin ligases (*Trim63* and *Fbxo32*), while increasing antioxidant enzyme levels and serum IL-15 in DMA. In tumor necrosis factor-α-stimulated L6 myotubes, QA reversed inflammation- and oxidative stress-induced gene changes, suppressed NF-ĸB activation, and downregulated protein degradation pathways mediated by FoxO3α. Furthermore, QA restored the expression of myogenesis-related genes and reactivated PI3K/Akt and mTOR/p70S6K/4EBP1 signaling pathways, enhancing protein synthesis. Collectively, our findings demonstrate that QA mitigates immobilization-induced muscle atrophy by modulating inflammation, oxidative stress, and key anabolic and catabolic signaling pathways. These results suggest that QA is a promising functional compound for preserving skeletal muscle health under conditions of disuse.

## 1. Introduction

Disuse-induced muscle atrophy by immobilization develops under conditions such as prolonged bed rest, limb immobilization, reduced physical activity, and exposure to microgravity [1]. These pathological states are characterized by a progressive loss of muscle mass and fiber number, along with a disturbance in the equilibrium between anabolic and catabolic processes [2]. Immobilization-induced disuse atrophy is closely associated with persistent inflammatory responses and excessive oxidative stress, which serve as pivotal drivers of skeletal muscle deterioration. Pro-inflammatory cytokines, including tumor necrosis factor-α (TNF-α) and interleukin-6 (IL-6), accelerate muscle wasting by stimulating the ubiquitin–proteasome pathway, thereby promoting excessive protein degradation and functional decline [3]. On the other hand, oxidative stress has emerged as another crucial contributor, as elevated reactive oxygen species (ROS) impair protein homeostasis through suppression of the mammalian target of rapamycin (mTOR)-mediated protein synthesis while simultaneously enhancing proteolysis via the ubiquitin–proteasome system [4,5]. Furthermore, excessive ROS exacerbates muscle wasting by activating nuclear factor kappa B (NF-κB)-mediated pro-inflammatory signaling cascade [6,7]. Collectively, these molecular alterations establish a vicious cycle of inflammation, oxidative stress, and proteolysis that accelerates the progression of disuse-induced atrophy. Accordingly, natural bioactive compounds with antioxidant and anti-inflammatory properties have attracted considerable attention as promising candidates for functional food ingredients aimed at preventing or mitigating immobilization-induced muscle atrophy.

**Figure 1:**
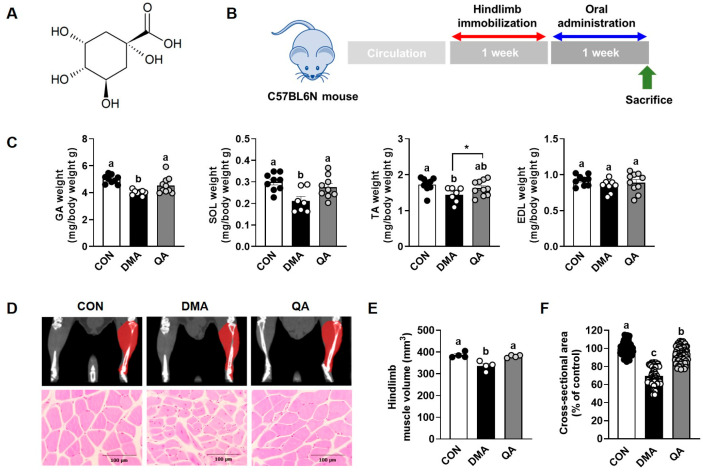
Protective effects of QA against the reduction in muscle weight and volume in DMA-induced mice. After inducing muscle atrophy through 1 week of immobilization, mice were administered saline or QA (50 mg/kg/day) orally for the subsequent week. (**A**) Structure of QA. (**B**) Experimental design. (**C**) The weights of GA, SOL, TA, and EDL. *n* = 9–10. (**D**,**E**) Representative micro-CT images showing muscle volume and (**F**) hematoxylin and eosin (H&E) images for myofiber size and quantification of CSA (% of control). *n* = 4–6. The results are expressed as mean values with their corresponding SEM. Groups marked with different letters were considered significantly different from one another (*p* < 0.05). Asterisk (*) indicates statistically significant differences between the two groups based on unpaired *t*-test results (*p* < 0.05). Each dot represents an individual mouse. Filled and open circles indicate data points from different experimental groups.

Quinic acid (QA, Figure 1A), also known as cyclohexanecarboxylic acid, is a naturally occurring organic acid widely distributed in dietary sources, including coffee beans, tea, carrots, and various fruits [8]. In addition to its natural abundance, QA can be obtained through the hydrolytic cleavage of chlorogenic acids, which are esters formed between QA and hydroxycinnamic acids [9]. Accumulating evidence has demonstrated that QA exerts diverse biological activities, including antioxidant, anti-inflammatory, anti-diabetic, anti-cancer, and antimicrobial effects [8]. Notably, QA has been shown to mitigate neuroinflammation in lipopolysaccharide-induced microglia and vascular inflammation in TNF-α-stimulated vascular smooth muscle cells, primarily through suppression of MAP kinase and NF-κB signaling pathways [10,11,12]. Furthermore, QA derivatives, such as 4,5-dicaffeoyl quinic acid, exhibited strong antioxidant effects by scavenging DPPH radicals, enhancing catalase and glutathione peroxidase activities, and protecting C6 glioma cells from tetrahydropapaveroline-induced cytotoxicity [8,13]. Beyond its well-established antioxidant and anti-inflammatory activities, QA has been shown to inhibit α-glucosidase activity and glucose transport in intestinal epithelial cells [14] and to activate AMP-activated protein kinase (AMPK) signaling in high-fat-diet-induced obese mice [15]. These findings suggest that the biological effects of QA may involve pleiotropic actions beyond its primary antioxidant and anti-inflammatory mechanisms. Compared with many plant-derived polyphenolic compounds that exhibit poor absorption or instability, QA and its acyl-quinic derivatives show moderate intestinal absorption and metabolic stability, resulting in detectable systemic metabolites and biological activity [16,17]. These pharmacokinetic advantages, along with QA’s ability to regulate inflammatory and metabolic pathways, underscore its translational potential as a promising candidate for preventing skeletal muscle atrophy. Collectively, these findings indicate that QA has considerable potential to prevent skeletal muscle atrophy through its antioxidant and anti-inflammatory activities. However, its direct role in muscle atrophy has not yet been elucidated. Therefore, in this study, we investigated whether QA could attenuate immobilization-induced muscle atrophy in C57BL/6N mice and further explored its underlying mechanisms by evaluating protein synthesis and degradation pathways in TNF-α-stimulated L6 myotubes.

## 2. Materials and Methods

### 2.1. Animal Study

Male C57BL/6N mice at age of 8 weeks (Samtaco, Gyeonggi-do, Republic of Korea) were maintained under standard animal room conditions (55 ± 5% relative humidity, 23 ± 2 °C temperature, and 12 h light-dark cycle) with ad libitum access to tap water and food throughout the experiment period in the Yonsei Laboratory Animal Research Center (YLARC; Seoul, Republic of Korea). After 1-week acclimation period, total 30 mice were randomly assigned to three groups (*n* = 10 per group): (I) CON (control), healthy mice without immobilization that received oral administration of saline as a vehicle; (II) DMA (disuse-induced muscle atrophy), mice subjected to hindlimb immobilization to induce disuse muscle atrophy and orally administered saline; and (III) QA (disuse + QA treatment), mice with disuse-induced muscle atrophy receiving oral administration of QA at 50 mg/kg/day. The sample size was estimated using G*Power software (version 3.1.9.4) for a one-way ANOVA design (effect size f = 0.59, α = 0.05, and power = 0.80). The calculation indicated that 27 mice (*n* = 9 per group) were required. To compensate for possible experimental loss, one additional mouse was included in each group, resulting in a total of 30 mice (*n* = 10 per group). Disuse-induced muscle atrophy was induced by immobilizing the right hindlimb for 1 week, while the left hindlimb remained non-immobilized to serve as an internal control, following a previously described procedure [18]. At the end of the immobilization period, the skin staples were removed from the hindlimb under anesthesia. Mice were orally administered either saline or QA (Sigma-Aldrich, St. Louis, MO, USA) once daily for 1 week. For the immobilization surgery, mice received intraperitoneal anesthesia with 325 mg/kg tribromoethanol (Sigma-Aldrich). The right hindlimb was stapled with one tine inserted into the foot and limb using the Acos 35W skin stapler (Sunmedix Co., Ltd., Gyeonggi-do, Republic of Korea). At the end of the treatment period, grip strength, treadmill performance, and muscle volume were measured, after which the mice were sacrificed under anesthesia by cardiac puncture. The non-immobilized control group served as a reference for baseline activity. Gastrocnemius (GA), soleus (SOL), tibialis anterior (TA), and extensor digitorum longus (EDL) muscles were harvested and snap-frozen in liquid nitrogen for gene and protein expression analysis or fixed in 10% formalin for histological analysis. To minimize potential confounding effects, cages were arranged in an alternating pattern across the experimental groups, and all treatments and measurements were conducted in a standardized sequence. Blinding was not feasible because the study required direct handling of the animals for daily oral administration, body weight monitoring, and behavioral testing. Humane endpoints were not specifically defined, as the experimental procedures were not anticipated to cause significant pain or distress. The mice health status was carefully monitored throughout the dosing period, and all experimental procedures were carried out in compliance with institutional animal care guidelines to minimize discomfort and stress. All animal experimental procedures were prudently reviewed and approved by the Institute of Animal Care and Use Committee (IACUC) of Yonsei University (Seoul, Republic of Korea) (Permit No.: IACUC-A-202012-1185-01).

### 2.2. Micro-Computed Tomography (Micro-CT) Imaging

Hindlimb muscle volumes were assessed with a small animal positron emission tomography/CT/single-photon emission tomography system (Siemens Inveon, Knoxville, TN, USA) at the Center for Evaluation of Biomaterials (Pohang Technopark, Pohang, Republic of Korea). CT images were analyzed using the Inveon Research Workplace software (version 4.2, Siemens Inveon, Knoxville, TN, USA).

### 2.3. Histological Analysis

TA muscles were fixed in 10% formalin solution (Junsei, Tokyo, Japan) and stained with hematoxylin and eosin (H&E). The stained sections were examined using a CK40 inverted microscope (Olympus, Tokyo, Japan) equipped with a T500 camera (eXcope, Daejeon, Republic of Korea). The cross-sectional area (CSA) of muscle fibers was quantified as the mean value using ImageJ software (version 1.47, National Institutes of Health, Bethesda, MD, USA).

### 2.4. Grip Strength Test

Grip strength was measured using a grip strength meter (Panlab, Barcelona, Spain) to assess forelimb and hindlimb strength of mice, as previously described [18]. Mice were held by the tail and gently pulled backward by the examiner, and the grip strength was recorded at the moment the mouse released its forepaws from the grid. At least five measurements were recorded for each mouse, and the average of these was used for statistical analysis.

### 2.5. Treadmill Test

Exercise capacity was assessed using a rodent treadmill system (LE8710MTS; Panlab, Barcelona, Spain). Mice were forced to run until exhaustion, which was defined as the inability to avoid the electric grid for ≥30 s. The time and distance to exhaustion were recorded for each mouse. The treadmill was set at 15 cm/s for the first 10 min and subsequently increased by 1 cm/s every min until a maximum speed of 35 cm/s was reached.

### 2.6. Enzyme-Linked Immunosorbent Assay (ELISA)

Serum samples were prepared by centrifugation of collected blood at 1300× *g* for 15 min, and the concentration of IL-15 was determined using a commercial ELISA kit (Merck Millipore, Burlington, MA, USA, manufactured in Germany) in accordance with the manufacturer’s protocol.

### 2.7. Cell Culture

L6 rat myoblasts (ATCC, Manassas, VA, USA) were cultured as previously described [19]. To induce differentiation, cells at approximately 70–80% confluence were switched from growth medium to differentiation medium composed of Dulbecco’s Modified Eagle’s Medium (HyClone, Logan, UT, USA) supplemented with 1% horse serum (Gibco, Gaithersburg, MD, USA) and 100 U/mL penicillin with 100 μg/mL streptomycin. The differentiation medium was replaced every other day for 6 days. Fully differentiated L6 myotubes were then treated with recombinant rat TNF-α (50 ng/mL; PeproTech, Rocky Hill, NJ, USA) for 24 h to induce skeletal muscle atrophy, with or without QA (20 or 40 μM). QA (Sigma-Aldrich) was dissolved in DMSO to prepare a 100 mM stock solution, aliquoted, and stored at −20 °C. Before each experiment, the stock was freshly diluted with culture medium to final concentrations of 20–40 µM, ensuring that the dimethyl sulfoxide concentration did not exceed 0.1% (*v*/*v*). According to the manufacturer’s information, QA is highly water-soluble and stable under standard laboratory conditions, and no precipitation or pH change was observed during treatment.

### 2.8. Reverse Transcription-Polymerase Chain Reaction (RT-PCR)

Total RNA was extracted from L6 myotubes and TA muscle tissues using TRIzol reagent (Takara Bio, Otsu, Japan). Complementary DNA synthesis and PCR amplification were performed with the GeneAmp^®^ PCR system 2700 (Applied Biosystems, Foster City, CA, USA) using Reverse Transcriptase Premix (Elpis-Biotech, Daejeon, Republic of Korea) and PCR premix (ELPIS-Biotech), as previously described. PCR products were separated on a 1.5% agarose gel, stained with Loading STAR dye (Dyne Bio, Seongnam, Republic of Korea), and visualized using a G:BOX EF imaging system (Syngene, Cambridge, UK).

### 2.9. Western Blot Analysis

The molecular mechanisms of the Akt/mTOR/forkhead box protein O3α (FoxO3α) signaling pathway were examined by Western blot analysis in L6 myotubes and TA muscles, as previously described [18]. Protein expression levels were detected using a G:BOX imaging system equipped with GeneSys software version 1.3.9.0 (Syngene, Cambridge, UK). The following antibodies were used: PI3K, phosphorylated PI3K (p-PI3K), Akt, phosphorylated Akt (p-Akt), mTOR, phosphorylated mTOR (p-mTOR), p70S6K, phosphorylated p70S6K (p-p70S6K), 4EBP1, phosphorylated 4EBP1 (p-4EBP1), and α-tubulin (Cell Signaling Technology, Beverly, MA, USA), as well as NF-κB (Santa Cruz Biotechnology, Santa Cruz, CA, USA). α-Tubulin was used as the loading control to normalize protein expression, as its level remained unchanged across all samples. Ponceau S staining (Thermo Scientific, Waltham, MA, USA) was performed prior to antibody incubation to confirm equal protein loading and transfer efficiency.

### 2.10. Statistical Analysis

Group differences were analyzed by one-way ANOVA followed by Tukey’s post hoc test, or by unpaired *t*-tests when appropriate. Statistical analyses were performed using GraphPad Prism version 10.0 (GraphPad Software, La Jolla, CA, USA). Outlier detection was performed with the GraphPad outlier calculator before conducting the statistical analyses. Data are presented as the mean ± standard error of the mean (SEM), and differences were considered statistically significant at *p* < 0.05.

## 3. Results

### 3.1. QA Restored the DMA-Induced Reductions in Muscle Weights Along with Muscle Fiber CSA

To assess the protective effects of QA on muscle atrophy, we employed a DMA-induced immobilization model in mice. Muscle atrophy was induced by 1 week of DMA-mediated immobilization, after which mice received oral saline or QA once daily for an additional week without immobilization (Figure 1B). Immobilization stress significantly reduced the weights of fore-type muscles, including GA, SOL, and TA, by 20.0, 27.6, and 16.3%, respectively, compared with the CON group (Figure 1C). Oral administration of QA significantly ameliorated these reductions, increasing GA, SOL, and TA weights by 13.3, 28.6, and 12.5%, respectively, relative to the DMA group. EDL muscle weight did not differ between groups. Consistently, the right hindlimb muscle volume was significantly lower in the DMA group, showing a 13.0% decrease compared to the CON group. However, QA supplementation attenuated this reduction by approximately 11.6%, maintaining muscle volume at a level similar to the CON group (Figure 1D,E). Since the CSA of skeletal muscle strongly correlates with muscle mass and reflects muscle fiber hypertrophy or atrophy, we further observed CSA changes to evaluate the effects of QA. The CSA of muscle fibers in the DMA group was 30.3% lower than that in the CON group, and this reduction was significantly restored by 23.0% following QA administration (Figure 1F).

### 3.2. QA Enhanced Grip Strength and Exercise Capacity in DMA-Induced Muscle Atrophy Mice

**Figure 2:**
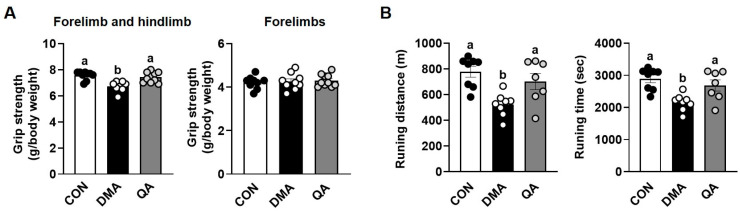
Improving effects of QA on grip strength and exercise endurance in DMA-induced mice. After inducing muscle atrophy through 1 week of immobilization, mice were administered saline or QA (50 mg/kg/day) orally for the subsequent week. (**A**) Grip strength of all four paws (forelimbs and hindlimbs combined) and of the forelimbs alone (two paws). (**B**) Running distance and time. *n* = 9–10. The results are expressed as mean values with their corresponding SEM. Groups marked with different letters were considered significantly different from one another (*p* < 0.05). Each dot represents an individual mouse. Filled and open circles indicate data points from different experimental groups.

Since QA administration significantly prevented immobilization-induced muscle mass loss, which is closely associated with muscle function, we subsequently evaluated grip strength and treadmill performance. Grip strength was measured for all four paws (forelimbs and hindlimbs combined) and for the forelimbs alone (two paws) to assess overall and limb-specific muscle function. Grip strength of the fore- and hindlimbs combined in the DMA group was significantly reduced by 15.5% compared with the CON group, whereas QA administration improved overall grip strength by 8.9% (Figure 2A). However, forelimb grip strength showed no significant differences among the groups, reflecting that immobilization was applied only to the hindlimb. Running distance and time, indicators of exercise capacity, were significantly reduced in the DMA group compared with the CON group by 33.0% and 25.4%, respectively (Figure 2B). However, QA administration markedly improved both parameters in the QA group by 34.7% and 24.4%, respectively.

### 3.3. QA Inhibited Muscle-E3 Ubiquitin Ligases Along with the Modulation of Inflammatory Cytokines and Antioxidant Enzymes in the TA Muscle of DMA-Induced Mice

**Figure 3:**
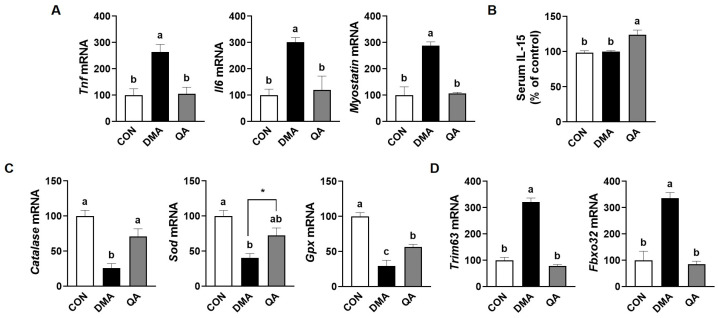
Effect of QA on the gene expression of pro-inflammatory cytokines, antioxidant enzymes, and E3 ubiquitin ligases in TA muscle of DMA-induced mice. After inducing muscle atrophy through 1 week of immobilization, mice were administered saline or QA (50 mg/kg/day) orally for the subsequent week. (**A**) mRNA expression levels of pro-inflammatory cytokines and myokines were analyzed by RT-PCR. (**B**) Serum levels of IL-5. (**C**) mRNA expression levels of antioxidant enzymes and (**D**) muscle-specific E3 ubiquitin ligases were analyzed by RT-PCR. *n* = 6. The results are expressed as mean values with their corresponding SEM. Groups marked with different letters were considered significantly different from one another (*p* < 0.05). Asterisk (*) indicates statistically significant differences between the two groups based on unpaired *t*-test results (*p* < 0.05).

Chronic inflammation and oxidative stress are critical factors that disturb the balance between protein synthesis and breakdown during muscle atrophy caused by immobilization [3]. Therefore, we next examined the gene expression levels of pro-inflammatory cytokines, antioxidant enzymes, and muscle-specific E3 ubiquitin ligases in the TA muscles of mice. The gene expression levels of *Tnf*, *Il6*, and *Myostatin* were significantly elevated in the DMA group compared to the CON group, whereas these increases were reduced by QA administration to levels similar to the CON group (Figure 3A). Serum IL-15, a myokine known to promote muscle growth and counteract atrophy, was significantly elevated by QA administration in immobilization stress-induced DMA mice, whereas no difference was observed between the CON and DMA groups (Figure 3B). The gene expression of antioxidant enzymes, including *Catalase*, *Sod*, and *Gpx*, was significantly increased by the immobilization stress in the DMA group, which were attenuated to levels of the CON group by QA administration (Figure 3C). Furthermore, disuse muscle atrophy by immobilization stress leads to significantly increase the gene expression of the muscle-specific E3 ubiquitin ligases, such as including muscle ring finger 1 (MuRF1; gene name *Trim63*) and muscle atrophy F-box (Atrogin-1; gene name *Fbxo32*), in DMA group compared to the CON group (Figure 3D).

### 3.4. QA Suppressed NF-κB-Driven Pro-Inflammatory Cytokines and Increased Antioxidant Enzymes in TNF-α-Induced L6 Myotube Atrophy

**Figure 4:**
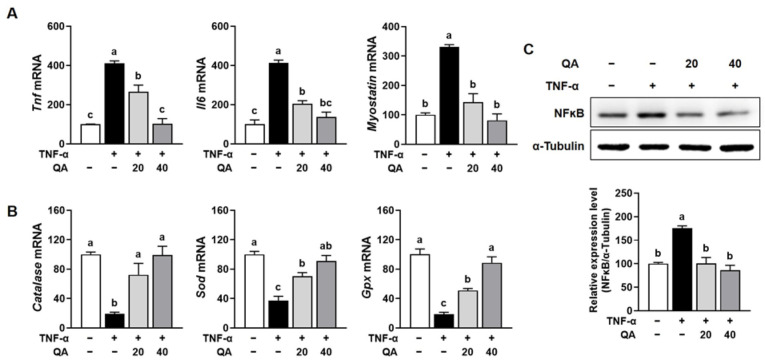
Effect of QA on the gene expression of pro-inflammatory cytokines and antioxidant enzymes in TNF-α-induced L6 myotube. L6 myotubes were exposed to TNF-α (50 ng/mL) in the presence or absence of QA (20 or 40 μM) for 24 h. (**A**) mRNA expression levels of pro-inflammatory cytokines, myokines, and (**B**) antioxidant enzymes were analyzed by RT-PCR. (**C**) The protein expression of NF-κB was analyzed by Western blotting. β-Actin and α-tubulin were used as internal controls for RT-PCR and Western blot analyses, respectively. The results are expressed as mean values with their corresponding SEM. Groups marked with different letters were considered significantly different from one another (*p* < 0.05).

We observed that QA significantly prevented disuse-induced muscle atrophy caused by immobilization stress in mice through the regulation of pro-inflammatory cytokines and antioxidant-related genes. To further investigate the underlying mechanism, we examined whether QA modulates protein degradation and synthesis pathways in TNF-α-induced muscle atrophy using differentiated L6 myotubes. Since immobilization-induced muscle wasting is closely associated with elevated inflammatory cytokines such as TNF-α, L6 myotubes were treated with TNF-α to mimic this catabolic condition. This approach allowed us to assess whether QA can attenuate TNF-α-induced myotube atrophy by regulating key molecular pathways involved in muscle proteostasis. TNF-α stimulation significantly increased the gene expression levels of *Tnf*, *Il6*, and *Myostatin* in L6 myotubes, which were completely restored to control levels by QA treatment (Figure 4A). The gene expression of antioxidant enzymes, such as *Catalase*, *Sod*, and *Gpx* was significantly suppressed by TNF-α stimulation in L6 myotubes, whereas QA treatment restored their expression in a dose-dependent manner to levels comparable with the CON (Figure 4B). Furthermore, TNF-α exposure markedly increased NF-κB protein expression in L6 myotubes, which was significantly restored to control levels by QA treatment (Figure 4C).

### 3.5. QA Attenuated Protein Degradation While Enhancing Protein Synthesis in TNF-α-Induced L6 Myotube Atrophy

**Figure 5:**
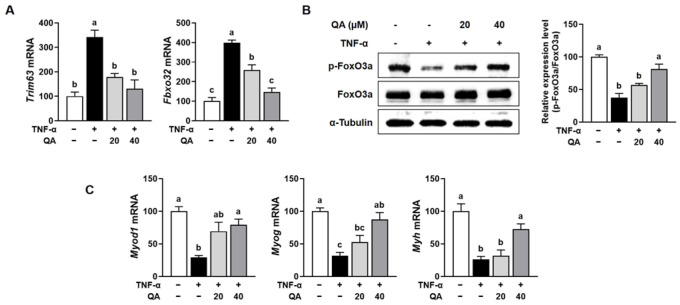
Effect of QA on protein degradation and myogenesis-related markers in TNF-α-induced L6 myotube. L6 myotubes were exposed to TNF-α (50 ng/mL) in the presence or absence of QA (20 or 40 μM) for 24 h. (**A**) mRNA expression levels of *Trim63* and *Fbxo32* were analyzed by RT-PCR. (**B**) The protein expression of FoxO3a phosphorylation was analyzed by Western blotting. (**C**) mRNA expression levels of *Myod1*, *Myog*, and *Myh* were analyzed by RT-PCR. β-Actin and α-tubulin were used as internal controls for RT-PCR and Western blot analyses, respectively. The results are expressed as mean values with their corresponding SEM. Groups marked with different letters were considered significantly different from one another (*p* < 0.05).

**Figure 6:**
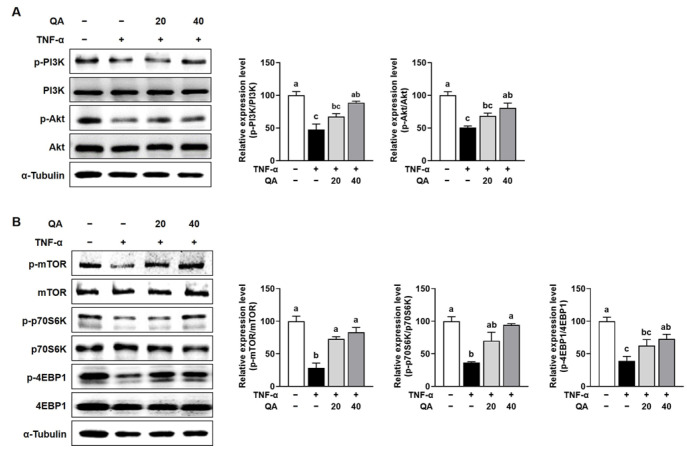
Effect of QA on protein synthesis-related markers in TNF-α-induced L6 myotube. L6 myotubes were exposed to TNF-α (50 ng/mL) in the presence or absence of QA (20 or 40 μM) for 24 h. (**A**) The protein expression of PI3K and Akt phosphorylation was analyzed by Western blotting. (**B**) The protein expression of mTOR, p70S6K, and 4EBP1 phosphorylation was analyzed by Western blotting. α-Tubulin was used as an internal control for Western blot analyses. The results are expressed as mean values with their corresponding SEM. Groups marked with different letters were considered significantly different from one another (*p* < 0.05).

Muscle atrophy is driven by two principal processes, reduced protein synthesis and elevated protein degradation [2]. Therefore, we examined the potential of QA to regulate these pathways in TNF-α-induced muscle wasting in L6 myotubes. The gene expression of muscle-specific E3 ubiquitin ligases, including *Trim63* and *Fbxo32*, was significantly increased by TNF-α stimulation in L6 myotubes, while QA treatment significantly attenuated these increases (Figure 5A). Also, QA treatment dose-dependently counteracted the TNF-α-induced increase in FoxO3α phosphorylation in L6 myotube (Figure 5B). The expression of myogenesis-regulating genes, including *Myod1*, *Myog*, and *Myh*, was significantly reduced by TNF-α exposure in L6 myotubes, but QA treatment restored their expression (Figure 5C). TNF-α stimulation in L6 myotubes reduced the protein expression of phosphorylated PI3K and Akt, but QA significantly restored their levels (Figure 6A). Continually, phosphorylation of mTOR, p70S6K, and 4EBP1 was markedly decreased in L6 myotubes stimulated with TNF-α, but QA treatment completely restored their phosphorylation level (Figure 6B).

## 4. Discussion

Skeletal muscle atrophy is a complex and debilitating condition marked by a progressive loss of muscle mass, strength, and function [20]. It occurs in a wide range of clinical and physiological contexts, including disuse, denervation, sepsis, cachexia, aging, and chronic inflammatory diseases [21]. Muscle atrophy not only compromises physical performance and quality of life but also increases the risk of morbidity and mortality, particularly in elderly and critically ill populations [22]. At the molecular and cellular level, muscle atrophy results from an imbalance between protein synthesis and protein degradation, favoring catabolism that drives muscle wasting [23]. Understanding the interplay between protein synthesis and degradation is crucial for elucidating the pathophysiology of muscle atrophy and for developing novel therapeutic interventions that aim to preserve or restore muscle mass [23,24]. Despite its clinical significance, there are no broadly approved pharmacological treatments that effectively prevent or reverse muscle wasting, nor improve muscle function. Current therapeutic strategies are limited and often associated with side effects. Therefore, there is a growing interest in identifying safe, naturally derived compounds that can modulate key molecular pathways involved in muscle degradation. In the present study, we investigated for the first time whether quinic acid, a naturally occurring compound with known antioxidant and anti-inflammatory properties, can effectively attenuate skeletal muscle atrophy induced by immobilization stress in vivo and TNF-α-stimulated myotubes in vitro. Disuse-induced muscle atrophy is characterized by disrupted homeostasis between anabolic and catabolic processes, largely driven by chronic inflammation and oxidative stress [25]. The observed decrease in muscle mass and CSA following disuse of muscle by immobilization was significantly mitigated by QA treatment. These morphological improvements translated into functional gains, as evidenced by restored grip strength and improved treadmill performance. Importantly, while QA restored forelimb and hindlimb grip strength, no significant improvement was observed in hindlimb grip strength. This differential effect may reflect regional variations in muscle susceptibility to immobilization or in QA bioavailability, warranting further investigation. Consistent with these results, phenolic compounds and polyphenols have been shown to preserve muscle mass and CSA and improve functional performance, including grip strength and exercise endurance in several mouse models. Chlorogenic acid treatment improved endurance exercise performance and increased grip strength in mice fed a high-fat diet, and significantly improved the strength of muscle in a resistance-trained rat model [26,27]. EGCG significantly exerted greater muscle mass than the controls, and showed a trend for increased muscle CSA in both 20-month-old Sprague-Dawley rats and in hindlimb suspension in senescent Fischer 344xBrown Norway inbred rats [28,29]. The interaction between inflammation and oxidative stress is the major contributor to muscle atrophy [30,31,32]. Mechanistically, the beneficial effects of QC on DMA in mice were associated with the suppression of pro-inflammatory cytokines and oxidative stress, the downregulation of muscle-specific E3 ubiquitin ligases, and the activation of key signaling pathways involved in protein synthesis. The immobilization model induced significant muscle wasting, accompanied by increased expression of pro-inflammatory cytokines (*Tnf* and *Il6*) and muscle atrophy-related genes (*Myostatin, Trim63*, and *Fbxo32*), as well as a compensatory increase in antioxidant enzyme gene expression (*catalase*, *Sod*, and *Gpx*). Notably, QA supplementation effectively reversed these pathological changes, suggesting that QA interrupts the vicious cycle of inflammation and oxidative stress that accelerates muscle loss. Several phenolic acids and polyphenols have been reported to have protective effects on muscle by regulating inflammation and oxidative stress signaling pathways. Caffeic acid phenethyl ester exerted protective effects against eccentric exercise-induced muscle injury in rats by inhibiting the activation of NF-κB and its downstream inflammatory genes such as cyclooxygenase-2, inducible nitric oxide synthase, monocyte chemotactic protein-1 and IL-1β [33]. Quercetin supplementation has been shown to reduce muscle mass and fiber size by inhibiting the genes involved in protein degradation and inflammation in muscle atrophy induced by obesity [34]. Resveratrol prevented reduced muscle mass and improved CSA by promoting protein synthesis, inhibiting proteolysis, and modulating inflammatory signaling in the atrophic muscle of the chronic kidney disease mouse model [35]. Low-molecular-weight peptide fractions derived from chicken embryo hydrolysates displayed pronounced radical-scavenging and anti-inflammatory effects, reducing oxidative injury and promoting tissue recovery [36]. Collectively, these results suggest that QA may have the potential to protect against muscle atrophy by regulating genes involved in inflammation, oxidative stress, and protein turnover during immobilization.

To understand the underlying mechanism of QA on muscle atrophy, we investigated the pathways involved in muscle atrophy, including inflammation, oxidative stress, protein degradation, and protein synthesis in TNF-α-treated L6 myotubes. Our in vitro findings using TNF-α-stimulated L6 myotubes further support the anti-inflammatory and antioxidative roles of QA. QA downregulated TNF-α-induced expression of *Tnf*, *Il6*, and Myostatin, while restoring antioxidant enzyme expression suppressed by inflammatory stress. These effects were accompanied by a reduction in NF-κB activation, a central mediator of inflammation and ROS production in muscle wasting. The suppression of NF-κB signaling by QA highlights its potential to break the inflammatory feedback loop that contributes to progressive muscle atrophy. Furthermore, QA inhibited TNF-α—induced expression of muscle-specific E3 ubiquitin ligases *Trim63* (MuRF1) and *Fbxo32* (Atrogin-1), key mediators of protein degradation via the ubiquitin-proteasome system. This suppression was associated with decreased phosphorylation of FoxO3α, a transcription factor that regulates the expression of E3 ligase genes and promotes catabolic processes. These findings suggest that QA inhibits proteolysis in part by modulating FoxO3α activity. QA restored the expression of myogenesis-related genes (*Myod1*, *Myog* and *Myh*), which were suppressed by TNF-α exposure. This finding implies a potential regenerative or muscle-maintaining role of QA under atrophic conditions. QA also activated the PI3K/Akt and mTOR/p70S6K/4EBP1 pathways, both of which are critical for promoting protein synthesis and muscle hypertrophy. These data indicate that QA not only suppresses muscle protein degradation but also enhances protein synthesis, contributing to the maintenance of muscle mass during disuse. In parallel with these results, ferulic acid regulated muscle type formation by increasing the expression of MyHC I, MyHC IIa and decreasing the mRNA abundance of MyHC IIb in C2C12 myotubes. The alteration of these genes was regulated via the Sirt1/AMPK signal pathway [37]. In the zebrafish model, ferulic acid upregulated anabolic MyH, myogenic MyoD and myogenin via regulation of zTOR (zebrafish target of rapamycin), p70S6K, and 4E-BP1 [38]. Coffee, rich in phenolic compounds, upregulated PGC-1α for promoting myogenic differentiation and myogenin expression via activation of the MKK3/6-p38 pathway and in C2C12 cells. Furthermore, coffee has been shown to promote muscle hypertrophy by activating the Akt/mTORC1 signaling pathway [39]. Taken together, our findings suggest that QA ameliorates immobilization-induced muscle atrophy through a dual mechanism: suppression of catabolic pathways mediated by inflammation and oxidative stress, and activation of anabolic signaling pathways that promote protein synthesis and muscle regeneration. These effects are likely mediated, at least in part, via modulation of the NF-κB/FoxO3α axis and the PI3K/Akt/mTOR pathway. Further experiments using siRNA or specific pathway inhibitors are warranted to clarify the causal involvement of the NF-κB/FoxO3α and PI3K/Akt/mTOR pathways in the protective effects of QA.

Immobilization-induced skeletal muscle atrophy results from chronic inflammation and oxidative stress [3]. Pro-inflammatory cytokines accelerate muscle protein degradation, and NF-κB activation increases muscle-specific E3 ubiquitin ligases that promote proteolysis [40,41]. In this study, QA alleviated TNF-α-induced myotube atrophy by promoting protein synthesis and suppressing degradation through the Akt/mTOR/FoxO3a pathway, thereby restoring anabolic-catabolic balance in muscle cells. The protective effects of QA were also associated with reduced cytokine expression and inhibition of MAP kinase and NF-κB signaling, along with strong antioxidant activity that enhanced cellular redox defenses [8,10,11,12,13]. These combined actions suggest that QA integrates inflammatory and oxidative pathways to maintain muscle integrity. Moreover, cross-talk among these signaling networks may amplify its efficacy. Activation of Nrf2 enhances antioxidant enzyme expression and limits NF-κB-driven inflammation, while inhibition of MAPK stabilizes redox homeostasis. This reciprocal regulation forms a feedback loop that strengthens resistance to muscle wasting. Collectively, the coordinated modulation of these pathways by QA represents a multi-targeted mechanism against immobilization-induced skeletal muscle atrophy.

Standardized brewing of commercial coffee (20 g/900 mL) has been reported to deliver approximately 27–95 mg of acyl-quinic acids per cup, indicating that a daily intake of several tens of milligrams of QA is achievable through regular coffee consumption [16,42,43]. Accordingly, the oral dose of 50 mg/kg/day used in this study was selected based on both dietary relevance and previously reported in vivo efficacy. Heikkilä et al., (2019) demonstrated that QA at 75 mg/kg/day enhanced insulin secretion without altering pancreatic insulin content [44], while Arya et al. (2014) reported increased antioxidant enzyme activity and no signs of toxicity up to 500 mg/kg [45]. In agreement, OECD toxicity predictions classify QA as non-toxic (LD_50_ > 2000 mg/kg), confirming a wide safety margin [46]. Although the reported oral bioavailability of QA varies between 1% and 3% depending on experimental conditions and analytical methods [16,17,42], a conservative estimate of 1% bioavailability was used for pharmacokinetic extrapolation. Based on this assumption, an oral dose of 50 mg/kg/day (≈1.25 mg for a 25 g mouse) would yield an approximate plasma concentration of 30–40 µM, assuming complete systemic distribution. This estimation corresponds closely to the 20–40 µM concentrations used in our in vitro experiments, supporting that the cell culture conditions reflect physiologically achievable exposure levels. Therefore, the selected oral dose and the in vitro range together provide a biologically meaningful and translationally relevant framework for evaluating the protective effects of QA against immobilization-induced muscle atrophy. Although this study has certain limitations, such as the short experimental duration, small sample size, and use of only male mice, these factors do not undermine the overall conclusions. Future studies incorporating pharmacokinetic evaluation and both sexes will further validate and expand the translational relevance of our findings. Additionally, evaluating the effects of QA under normal physiological conditions would also provide valuable insight into its intrinsic actions on skeletal muscle beyond its protective role in immobilization-induced atrophy. Given that QA has been reported to possess antioxidant and anti-inflammatory properties even under physiological conditions [8,10,11,12,13], future studies will include a QA-only group to further elucidate its potential role in promoting muscle homeostasis and function under normal conditions.

## 5. Conclusions

In summary, the present study demonstrated that QA attenuates skeletal muscle atrophy in both immobilized hindlimb muscles of C57BL/6N mice and TNF-α-stimulated L6 myotubes. QA administration restored muscle strength and mass by increasing muscle volume and myofiber CSA. At the molecular level, QA suppressed the expression of pro-inflammatory cytokines and E3 ubiquitin ligases, while enhancing antioxidant enzyme activity and serum IL-15 levels. Furthermore, QA reactivated PI3K/Akt/mTOR signaling and inhibited NF-κB/FoxO3α-mediated protein degradation, thereby improving protein homeostasis and promoting myogenesis in both in vivo and in vitro models. Collectively, these findings suggest that QA is a promising candidate for the development of functional foods or nutraceuticals designed to preserve skeletal muscle mass and function under conditions of immobilization or disuse. Its regulatory effects on inflammation and oxidative stress offer a comprehensive strategy to combat muscle wasting and enhance recovery. Further studies using pathway-specific inhibitors, siRNA, and long-term evaluations are warranted to confirm the causal mechanisms and translational potential of QA.

## Data Availability

The original contributions presented in the study are included in the article; further inquiries can be directed to the corresponding authors.

## Abbreviations

The following abbreviations are used in this manuscript:

DMA	disuse-induced muscle atrophy
EDL	extensor digitorum longus
FoXO3α	forkhead box protein O3α
GA	gastrocnemius
IL	interleukin
mTOR	mammalian target of rapamycin
MuRF1	muscle ring finger 1
NF-κB	nuclear factor kappa B
PI3K	phosphatidylinositol 3-kinase
p-4EBP1	phosphorylated 4EBP1
p-Akt	phosphorylated Akt
p-mTOR	phosphorylated mTOR
p-PI3K	phosphorylated PI3K
p-p70S6K	phosphorylated p70S6K
QA	quinic acid
ROS	reactive oxygen species
SOL	soleus
TA	tibialis anterior
TNF-α	tumor necrosis factor-α
